# *Escherichia coli* harbouring *strAB* with reduced susceptibility towards gentamicin and amikacin: a single centre study from India

**DOI:** 10.1099/acmi.0.000446

**Published:** 2022-10-27

**Authors:** Jayalaxmi Wangkheimayum, Prynali Phonglo, K. Melson Singha, Debadatta Dhar Chanda, Amitabha Bhattacharjee

**Affiliations:** ^1^​ Department of Microbiology, Assam University, Silchar, India; ^2^​ Department of Microbiology, Silchar Medical College and Hospital, Silchar, India

**Keywords:** aminoglycoside, *Escherichia coli*, ESBL, StrAB

## Abstract

In this study we report the presence of streptomycin resistance gene *strAB* within clinical isolates of *Escherichia coli* where streptomycin is not used to treat Gram-negative infections. In total, 135 *E. coli* isolates were obtained for the study. PCR based detection of *strAB* was performed in the study isolates followed by assessment of horizontal transferability. Cloning of *strAB* was done in laboratory strain *E. coli* DH5α. Pre-cloning and post-cloning susceptibility of the strain was done for assessment of acquired resistance. Among tested isolates, 89 were found to harbour *strAB* and it was encoded within a IncI1 type plasmid. Cloning experiments revealed the *strAB* gene showed unusual non-susceptibility towards amikacin and gentamicin. The study highlighted that *strAB,* which has a role in streptomycin resistance, may also have a role in reduced susceptibility towards gentamicin and amikacin within a clinical setting.

## Introduction

Resistance to streptomycin was identified in 1945 by Waksman *et al*. and can be conferred through chromosomal mutations, thereby altering the ribosomal binding site of streptomycin [1]. It can also be conferred by enzymes which inactivate the molecule at one of the two sites through phosphorylation or adenylation [2]. *StrAB* encodes streptomycin-inactivating enzymes which are extensively distributed in both Gram-negative and Gram-positive bacteria. They are often located on broad-host-range plasmids in clinical bacterial isolates, and are frequently associated with the *sulII* gene that encodes a sulphonamide-resistant alternative dihydropteroate synthase [3, 4]. These genes are reported to be associated with transposons Tn*5393* [5]. They are part of the *aadA* gene cassette which is responsible for aminoglycoside resistance, especially streptomycin and spectinomycin [6]. Over the last 30 years, *strAB* genes have been reported worldwide in pathogens of plants, humans and animals [4]. However, clinical use of streptomycin is no longer practiced in hospitals for treating Gram-positive and Gram-negative infections where amikacin and gentamicin are predominantly being prescribed. Propagation and maintenance of *strAB* is reported to be in clinical settings and therefore it is of interest to understand if this resistance determinant has any role towards other aminoglycoside antibiotics.

Thus, the present study investigates the presence of *strAB* among clinical isolates, and its transmission dynamics, along with its spectrum of resistance towards other aminoglycoside antibiotics.

## Methods

### Bacterial isolates

A total of 135 non-duplicate isolates of *Escherichia coli* were obtained spanning 12 months (March 2019 to February 2020) from patients who were admitted to different wards or attended outpatient departments of Silchar Medical College and Hospital, Silchar, India. Samples were taken as a part of standard care and day-to-day routine sampling. The samples were urine (*n*=87), pus (*n*=24), wound swab (*n*=19) and aspirate (*n*=5). Cysteine-lactose-electrolyte-deficient agar (CLED) and blood agar was used for isolation. The isolates were identified by the VITEK two compact sub-system (BioMérieux, France).

### Antimicrobial susceptibility testing

Minimum inhibitory concentration (MICs) of the study isolates against gentamicin, kanamycin, amikacin, streptomycin, tobramycin, and netilmicin (Hi-Media, India) were determined by agar dilution method. For determination of susceptibility against other groups of antimicrobials, Kirby Baur disc diffusion method was used. Antibiotics tested were imipenem (10 µg), cefepime (30 µg), aztreonam (30 µg), cefotaxime (30 µg), ceftazidime (30 µg), ceftriaxone (30 µg), cotrimoxazole (25 µg), ciprofloxacin (5 µg), and streptomycin (10 µg) (Hi-Media, India). Results were interpreted as per Clinical and Laboratory Standards Institute (CLSI) guidelines 2017 [5]. *Escherichia coli* ATCC 25922 was used as a negative control.

### PCR based detection

Amplification of *strAB* was done using primer pairs *strAB*-forward 5′TATCTGCGATTGGACCCTCTG3′ and *strAB*- reverse 5′CATTGCTCATCATTTGATCGGCT3′ [6] for all the study isolates. The reaction was performed under the following conditions: initial denaturation at 95 °C for 2 mins, denaturation at 95 °C for 30 secs, annealing at 54 °C for 40 secs, extension at 72 °C for 1 min and a final extension at 72 °C for 7 mins. A previously confirmed laboratory isolate was used as a positive control. PCR products were separated by gel electrophoresis on 1 % agarose gel. Amplified products were sequenced by Sanger’s method.

### Pre-cloning susceptibility of *E. coli* DH5α

The pre-cloning antibiotic susceptibility testing of *E. coli* DH5α was performed to assess susceptibility towards different antibiotics. The antibiotics included were all those mentioned above in the susceptibility testing section. Results were interpreted as per CLSI guidelines 2017 [7].

### Cloning of *strAB*

To understand role of *strAB* towards other aminoglycoside antibiotics, cloning was done in *E. coli* DH5α, which is devoid of any aminoglycoside resistance genes. For the cloning of *strAB*, primers were designed (forward 5′GCTTGGTGATAACGGCAATTCC3′) and reverse-5′ATGTAAGGCCTTTGAATAAGAC3′) from the flanking region of *strAB* to amplify the whole gene including the promoter region. Ten isolates were selected randomly for amplification of the whole *strAB* gene. The amplified product was purified using MinElute PCR Purification Kit (Qiagen, Hilden, Germany) and was ligated into pGEM-T Vector (Promega, Madison, USA). Transformation was done using the laboratory strain *E. coli* DH5α by the heat-shock method. The recipient cell i.e., *E. coli* DH5α (in 0.1 M CaCl_2_, 10 % glycerol) and 10 µl of extracted plasmid (HiPurA Plasmid DNA Miniprep Purification kit, Hi-Media, India) from *strAB* harbouring isolates were mixed and incubated in ice for 30 min. The mixture was subjected to heat-shock at 42 °C for 40 s and it was immediately placed in ice for 10 min. Further, 500 µl of SOC solution was added and incubated for 2 h. The mixture was pelleted and spread over Luria Bertani agar containing 100 µg ml^−1^ of ampicillin. *E. coli* DH5α, without performing transformation, was also spread over a screened agar plate which acted as a control. Transformants were selected by blue-white screening in the presence of X-gal, IPTG, and 100 µg ml^−1^ of ampicillin in the screening LB agar. A PCR assay targeting *strAB* was performed to confirm the successful cloning of *strAB* within the host. The clones were further subjected to susceptibility testing against the previously mentioned aminoglycoside group of antibiotics by Kirby Bauer disc diffusion method. The overnight culture was diluted with normal saline and the turbidity was adjusted to MacFarland standard 0.5. A Meuller Hinton agar plate was used and the plates were incubated for 16 h. Three replicates of the clone were tested. The results were interpreted as per CLSI guidelines 2017.

### Transferability assay

Plasmids encoding *strAB* gene were extracted by QIAprep Spin Miniprep kit (Qiagen, Germany) as per manufacturer’s instructions. Isolated plasmids were further subjected to transformation assay by the heat-shock method using *Escherichia coli* DH5α as the recipient. Transformants were selected on the Luria Bertani agar (Hi-Media, Mumbai, India) containing 4 µg ml^−1^ of streptomycin. Conjugation assay was performed using *Escherichia coli* harbouring *strAB* as a donor and azide resistant *Escherichia coli* J53 as a recipient. Mating was performed where both the donor and recipient cells were cultured in LB broth (Hi-Media) by agitation until it attained optical density at 600 nm (OD_600_) of 0.8–0.9. Cells were mixed at a ratio of 1 : 5 donor-to-recipient, incubated for 2 h and the transconjugant was selected on LB agar (Hi-Media, Mumbai, India) containing 4 µg ml^−1^ of streptomycin and 100 µg ml^−1^ of sodium azide.

### PCR based replicon typing

Plasmids encoding the resistance determinant were characterized by PCR based replicon typing to identify the different incompatibility (Inc) groups viz. FIA, FIB, FIC, HI1, HI2, I1/IY, L/M, N, P, W, T, A/C, K, B/O, X, Y, F, and FIIA by performing five multiplex PCR and three simplex PCR [8].

### Detection of other co-existing aminoglycoside modifying enzymes (aminoglycoside acetyltransferase, amninoglycoside adenyltransferase and aminoglycoside phosphotransferase)

For amplification and characterization of all the co-existing aminoglycoside modifying enzyme genes (AMEs) two multiplex PCR assays were performed targeting various AME genes viz.; *ant(2″)-Ia, ant(3″)-I, ant(4′)-Ia, aac(3)-I, aac(3)-IIc, aac(6′)-Ib, aac(6′)-II*, genes viz.; *aph (2′)-Ib, aph (2′)-Ic, aph (2')-Id, aph (3')- IIb, aph (3')-I, aph (3′)-IIIa, aph (3')-Via,* and *aph (4)-Ia* (Table S1, available in the online version of this article). The PCR mixture was composed of 12.5 µl Go Taq Green Master mix (Promega, Madison, USA) with 10 pmol of each primer and ~100 ng DNA template. The reaction condition was described previously [9].

### Detection of extended spectrum beta-lactamases and carbapenemases

For amplification and characterization of *bla*_ESBL_, *bla*_TEM_, *bla*_CTX-M_, *bla*_SHV_, *bla*_PER_, *bla*_GES_, and *bla*_VEB_ (Table S2) were targeted. Reactions were run under the following conditions: initial denaturation at 94 °C for 5 mins, 33 cycles of 94 °C for 35 s, 51 °C for 1 min, 72 °C for 1 min and the final extension at 72 °C for 7 mins. For amplification and characterization of the carbapenem-resistance gene; *bla*_KPC_, *bla*_NDM_, *bla*_VIM_, *bla*_IMP_, *bla*_OXA-23_, *bla*_OXA-48_, and *bla*_OXA-58_ (Table S2) were targeted. Reactions were performed under the following conditions: initial denaturation at 94 °C for 10 min; 30 cycles of 94 °C for 40 s, 55 °C for 40 s and 72 °C for 1 min, and a final elongation step at 72 °C for 7 mins.

### Typing of the isolates by ERIC-PCR

The heterogeneity of the streptomycin-resistant isolates was determined by enterobacterial repetitive intergenic consensus (ERIC) PCR using primers ERIC-F (5′ATGTAAGCTCCTGGGGATTCAC-3′) and ERIC-R (5′AAGTAAGTGACTGGGGTGAGCG-3′) [9], and the banding patterns were analysed by agarose gel electrophoresis.

## Results

### Susceptibility testing

Antibiotic susceptibility testing showed imipenem was the most active (85 %; *n*=76) followed by cefepime (81 %; *n*=72), aztreonam (81 %; *n*=72), ceftazidime (79 %; *n*=71), cefotaxime (78 %; *n*=70), ceftriaxone (78 %; *n*=70), ciprofloxacin (55 %; *n*=49), and cotrimoxazole (41 %; *n*=37). MIC results showed that 50 % of the isolates showed an inhibition value in the range of intermediate (amikacin, netilmicin and kanamycin) to resistant (gentamicin and tobramycin) towards aminoglycoside antibiotics (Table 1). Isolates devoid of carbapenemases (*n*=76) and extended spectrum beta-lactamases (*n*=72) were sensitive to third generation cephalosporins and carbapenems. Among 135 isolates 121 (89.6 %) of them were resistant to at least one of the aminoglycosides tested; however, streptomycin resistance was found in all of them (*n*=121).

**Table 1.** MIC_50_ and MIC_90_ value of 89 isolates of *E. coli* that were harbouring *strAB* against different aminoglycoside antibiotics

Antimicrobial agents	MIC_50_ (µg ml^−1^)	MIC_90_ (µg ml^−1^)
Gentamicin	16	512
Tobramycin	32	512
Amikacin	32	512
Netilmicin	16	128
Kanamycin	32	512

### PCR detection of *strAB* and co-carriage of other resistance genes

Eighty-nine isolates harboured the *strAB* gene. Isolates carrying *strAB* were found co-harbouring aminoglycoside modifying enzyme genes in 58 isolates. Aminoglycoside phospho transferase types were common i.e. *aph (2')-Ib* (*n*=18), *aph (4)-Ia* (*n*=8), *aph (3')-IIb* (*n*=7), *aph (3')-I* (*n*=6), and *aph (3')-IIIa* (*n*=3) followed by aminoglycoside adenyl transferase types i.e. *ant(3″)-I* (*n*=6), *ant (4′)-Ia* (*n*=5) and *ant (2″)-Ia* (*n*=2) and variants of aminoglycoside acetyl transferase viz., *aac (3)-IIc* (*n*=2) and *aac (6′)-Ib* (*n*=1) (Fig. S1). Co-existing extended spectrum beta-lactamase was observed among 17 isolates, of which *bla*_CTX-M_ was common (*n*=11), followed by *bla*_TEM_ (*n*=3), *bla*_GES_ (*n*=1), *bla*_PER_ (*n*=1), and *bla*_SHV_(*n*=1). A total of 13 isolates were co-harbouring carbapenemases, of which 10 were carrying *bla*_NDM-1_, and four had *bla*_VIM_ (Table S3).

### Cloning of *strAB*

Cloning was performed to assess the role of *strAB* towards other aminoglyside antibiotics as the resistance determinant is only known for streptomycin resistance. Pre-cloning susceptibility testing showed that *E. coli* DH5α was susceptible to all of the aminoglycoside group of antibiotics tested. *StrAB* clones were selected by blue-white screening and PCR confirmed the presence of *strAB* within the host. All the clones were found to be streptomycin resistant. Additionally, all ten clones showed an intermediate-range, i.e., non-susceptible towards gentamicin, tobramycin, and amikacin (Table S5).

### Transferability of *strAB* and ERIC typing

Plasmids isolated from all the *strAB*-bearing isolates could be selected in the transformants when grown in media containing 4 µg ml^−1^ streptomycin, and the presence of a resistance gene within the plasmid was confirmed by PCR. Plasmids encoding *strAB* were also found to be conjugatively transferable for all the cases. Further, PCR based replicon typing confirmed that plasmids harbouring *strAB* were of the Inc I1 type. Enterobacterial repetitive intergenic consensus (ERIC) PCR showed 49 different haplotypes of *E. coli* that were harbouring the *strAB* gene.

## Discussion

Streptomycin has limited clinical usage due to hypersensitive reactions and currently being prescribed to treat multidrug resistant tuberculosis [10]. It is at the forefront for therapeutic use in animals and plants, and in additional growth promotion activity, making it very important for use in veterinary medicine [5]. Few studies have reported the presence of *strAB* within isolates that are obtained from animal and environmental sources, and a previous study reported the linkage of the *strA-strB* streptomycin-resistance genes with Class one integron sequences on pSTR1, which is isolated from multiple antibiotic-resistance plasmids from *Shigella flexneri* [11, 12]. *StrAB* and *aadA* antibiotic resistance cassette structures (ARCs) were identified and significantly associated with cattle isolates [13]. In an earlier study, 84 % of streptomycin resistant *Salmonella enterica* isolates from an animal origin were carrying *strAB* [5]. In another study, 66 % of streptomycin resistant *E. coli* were found to harbour *strAB,* which were isolated from meat samples [6]. Similarly, in the present study we have observed a 66 % occurrence of *strAB* in clinical isolates. The present study underscores the presence of a single Inc-type plasmid responsible for expression of the *strAB* gene through lateral gene transfer. Considering the scenario of an expansion of *strAB* from agriculture, animals to humans and vice versa, a holistic approach is needed to detect the resistance determinant and protect against further dissemination. However, the major limitation of this study is the lack of sequence data and genomic context which could support phenotypic observations. ERIC typing showed the presence of 49 haplotypes carrying *strAB*. This indicates that this resistance determinant has expanded into diverse haplotypes through lateral transfer in the study setting.

For the first time, this study observed that *strAB* has a role in reduced susceptibility towards clinically relevant aminoglycosides, i.e., gentamicin, amikacin, and tobramycin, which poses a potential threat in clinical settings. Therefore, further study with respect to whole genome analysis is required to understand this less recognized resistance gene and its role in aminoglycoside resistance in hospital and community environments.

## Supplementary Data

Supplementary material 1
