# Supplementary material for: Escherichia coli harbouring strAB with reduced susceptibility towards gentamicin and amikacin: a single centre study from India
Source: Access Microbiol. 2022 Oct 27;4(10):acmi000446. doi: 10.1099/acmi.0.000446 (PMC9675172; doi:10.1099/acmi.0.000446)
Supplement: Supplementary material 1 [file acmi-4-446-s001.pdf]

Supplementary Table S1: AMEs primer sequence used in the study

Sl.no	Target	Primer pairs	Sequence	Amplified product size (bp)	Reference
1	*ant(2")-Ia*	Forward	5’-GCTCACGCAACTGGTCCAGA-3’	719	Pak-Leung et al; 2016 [7]
		Reverse	5’-GGCACGCAAGACCTCAACCT-3’		
2	*ant(3")-I*	Forward	5’-TGATTTGCTGGTTACGGTGAC-3’	284	Pak-Leung et al; 2016 [7]
		Reverse	5’-CGCTATGTTCTCTTGCTTTTG-3’		
3	*ant(4')-Ia*	Forward	5’-CTGCTAAATCGGTAGAAGC-3’	172	Pak-Leung et al; 2016 [7]
		Reverse	5’-CAGACCAATCAACATGGCACC-3’		
4	*aac(3)-I*	Forward	5’-TTACGCAGCAGCAACGATGT-3’	402	Pak-Leung et al; 2016 [7]
		Reverse	5’-GTTGGCCTCATGCTTGAGGA-3’		
5	*aac(3)-IIc*	Forward	5′-ACGCGGAAGGCAATAAGGA-3′	854	Pak-Leung et al; 2016 [7]
		Reverse	5′-TAACCTGAAGGCTCGCAAGA-3′		
6	*aac(6')-Ib*	Forward	5′-CATGACCTTGCGATGCTCTA-3′	490	Pak-Leung et al; 2016 [7]
		Reverse	5′-GCTCGAATGCCTGGCGTCTT-3′		
7	*aac(6')-II*	Forward	5′-TTCATGTCCGCCAGCACCCC-3′	178	Pak-Leung et al; 2016 [7]
		Reverse	5′-GACTCTTCCGCCATCGCTCT-3′		

8	*aph(2")-Ib*	Forward	5′-CTTGGACGCTGAGATATATGAGCAC-3′	867	Pak-Leung et al; 2016 [7]
		Reverse	5′-GTTTGTAGCAATTCAGAAACACCCTT-3′		
9	*aph(2")-Ic*	Forward	5′-CCACAATGATAATGACTCAGTTCCC-3′	444	Pak-Leung et al; 2016 [7]
		Reverse	5′-CCACAGCTTCCGATAGCAAGAG-3′		
10	*aph(2')-Id*	Forward	5′-GTGGTTTTTACAGGAATGCCATC-3′	641	Pak-Leung et al; 2016 [7]
		Reverse	5′-CCCTCTTCATACCAATCCATATAACC-3′		
11	*aph(3')-I*	Forward	5′-ATGTGCCATATTCAACGGGAAACG-3′	816	Pak-Leung et al; 2016 [7]
		Reverse	5′-TCAGAAAAACTCATCGAGCATCAA-3′		
12	*aph(3')-IIb*	Forward	5’-ATGCATGATGCAGCCACCTCC-3′	804	Pak-Leung et al; 2016 [7]
		Reverse	5′-CTAGAAGAACTCGTCCAATAGCCT-3′		
13	*aph(3')-IIIa*	Forward	5′-GGCTAAAATGAGAATATCACCGG-3′	278	Pak-Leung et al; 2016 [7]
		Reverse	5′-CTTTAAAAAATCATACAGCTCGCG-3′		
14	*aph(3')-VIa*	Forward	5′-ATACAGAGAGACCACCATACAGT-3′	234	Pak-Leung et al; 2016 [7]
		Reverse	5′-GGACAATCAATAATAGCAAT-3′		
15	*aph(4)-Ia*	Forward	5′-CTGAACTCACCGCGACGTCT-3′	977	Pak-Leung et al; 2016 [7]
		Reverse	5′-TCCACTATCGGCGAGTACTT-3′		

Supplementary Table S2: Oligonucleotides used as primers in the study

Target	Primer pairs	Sequences	Product size	Reference
StrAB	StrAB-F	5'TATCTGCGATTGGACCCTCTG3'	538	Sunde et al; 2005 [4]
	StrAB-R	5'CATTGCTCATCATTTGATCGGCT3'		
StrW	StrW-F	5'GCTTGGTGATAACGGCAATTCC3'	1115	This study
	StrW-R	5'ATGTAAGGCCTTTGAATAAGAC3		
TEM	TEM-F	5'ATGAGTATTCAACATTTCCG3'	867	Bert et al; 2002 [12]
	TEM-R	5'CTGACAGTTACCAATGCTTA 3'		
SHV	SHV-F	5'AGGATTGACTGCCTTTTTG 3'	392	Colom et al; 2003 [13]
	SHV-R	5'ATTTGCTGATTTCGCTCG 3'		
CTX-M	CTX-M-F	5'CGCTTTGCGATGTGCAG 3'	550	Lee et al; 2005 [14]
	CTX-M-R	5'ACCGCGATATCGTTGGT 3'		
PER	PER-F	5'AAT TTG GGC TTA GGG CAG AA 3'	920	Lee et al; 2005 [14]
	PER-R	5'ATG AAT GTC ATT ATA AAA GC 3'		
GES	GES-F	5'AGTCGGCTAGACCGGAAAG 3'	863	Lee et al; 2005 [14]
	GES-R	5'TTTGTCCGTGCTCAGGAT 3'		
VEB	VEB-F	5'CATTTCCCGATGCAAAGCGT 3'	650	Lee et al; 2005 [14]
	VEB-R	5'CGAAGTTTCTTTGGACTCTG 3'		
KPC	KPC-F	5'CATTCAAGGCTTTCTTGCTGC3'	538	Nass et al; 1994 [15]
	KPC-R	5'ACGACGGCATAGTCATTTGC3'		
OXA-23	OXA-23-F	5'GATCGGATTGGAGAACCAGA3'	501	Woodford et al; 2004 [16]
	OXA-23-R	5'ATTTCTGACCGCATTTCCAT3'		
OXA-48	OXA-48-F	5'GATTATCGGAATGCCTGCGG3'	845	Cuzon et al; 2013 [17]
	OXA-48-R	5'CTACAAGCGCATCGAGCATCA3'		
OXA-58	OXA-58-F	5'CGATCAGAATGTTCAAGCGC3'	529	Poirel et al; 2005 [18]
	OXA-58-R	5'ACGATTCTCCCCTCTGCGC3'		

VIM	VIM-F	5'GATGGTGTTTGGTCGCATA3'	390	Tsakris et al; 2009 [19]
	VIM-R	5'CGAATGCGCAGCACCAG3'		
IMP	IMP-F	5'TTGACACTCCATTTACDC3'	139	Ito et al; 1995 [20]
	IMP-R	5'GATYGAGAATTAAGCCACYCT3'		
NDM	NDM-F	5'GGGCAGTCGCTTCCAACGGT3'	476	Yong et al; 2009 [21]
	NDM-R	5'GTAGTGCTCAGTGTCGGCAT3'		

Supplementary Table S3: Distribution of co-existing resistance genes within *E.coli* isolates harbouring *strAB*

Organism ID	Aminoglycoside modifying enzyme genes	Carbapenemase genes	ESBL genes
JW1	-	bla_{NDM}	-
JW4	-	-	bla_{CTX-M}
JW9	*aph(3´)-I*	-	-
JW12	*aph(3´)-I*	-	-
JW15	*aph(2´´)-Ib*	-	-
JW18	-	-	bla_{SHV}
JW21	-	-	bla_{CTX-M}
JW24	-	bla_{VIM}	-
JW29	*ant(2´´)-Ia*	-	-
JW32	*aph(4´)-Ia*	-	-
JW34	-	-	bla_{CTX-M}
JW38	*aac(3)-IIc*	-	-
JW40	-	bla_{NDM}	-
JW45	*aac(3)-IIc*	-	-
JW49	*aph(3´)-IIb*	-	-
JW56	*aph(4´)-Ia*	-	
JW61	-	bla_{NDM}	-
JW64	*ant(2´´)-Ia*	-	-
JW69	-	-	bla_{TEM}
JW72	*aph(2´´)-Ib*	-	-
JW78	*aac(3)-IIc*	-	-
JW82	*aph(3´)-IIb*	-	-
JW87	-	-	bla_{CTX-M}
JW93	-	bla_{VIM}	-

JW101	*aph(3´)-IIb*	-	-
JW105	*ant(4´)-Ia*	-	-
JW108	-	-	*bla*$_{\text{PER}}$
JW117	*aac(6´)-Ib*	-	-
JW123	*ant(3´´)-I*	-	-
JW128	*aph(2´´)-Ib*	-	-
JW132	-	-	*bla*$_{\text{TEM}}$
JW137	-	*bla*$_{\text{NDM}}$	-
JW139	-	-	*bla*$_{\text{CTX-M}}$
JW142	*aph(3´)-IIb*	-	-
JW147	-	-	*bla*$_{\text{CTX-M}}$
JW152	*aph(2´´)-Ib*	-	-
JW158	-	-	*bla*$_{\text{CTX-M}}$
JW161	-	-	*bla*$_{\text{GES}}$
JW164	*aph(4´)-Ia*	-	-
JW172	-	*bla*$_{\text{NDM}}$	-
JW188	*ant(3´´)-I*	-	-
JW191	-	*bla*$_{\text{NDM}}$	-
JW219	*aph(2´´)-Ib*	-	-
JW226	*aph(4´)-Ia*	-	-
JW245	*ant(4´)-Ia*	-	-
JW249	*aph(3´´)-IIIa*	-	-
JW255	*aph(3´´)-IIIa*	-	-
JW267	-	*bla*$_{\text{VIM}}$	-
JW273	*aph(2´´)-Ib*	-	-
JW280	*aph(2´´)-Ib*	-	-
JW296	-	-	*bla*$_{\text{TEM}}$
JW310	-	*bla*$_{\text{NDM}}$	-

JW345	*aph(3´)-IIb*	-	-
JW323	*aph(2´´)-Ib*	-	-
JW380	*aph(3´´)-IIIa*	-	-
JW395	*aph(2´´)-Ib*	-	-
JW411	*aph(2´´)-Ib*	-	-
JW427	-	bla_{NDM}	-
JW429	*ant(3´´)-I*	-	-
JW486	*aph(2´´)-Ib*	-	-
JW512	*aph(4´)-Ia*	-	-
JW558	-	-	$bla_{\mathrm{CTX\text{-}M}}$
JW591	*ant(4´)-Ia*	-	-
JW618	*aph(2´´)-Ib*	-	-
JW636	*aph(2´´)-Ib*	-	-
JW648	*aph(2´´)-Ib*	-	-
JW661	*aph(3´)-IIb*	-	-
JW673	-	-	$bla_{\mathrm{CTX\text{-}M}}$
JW682	*aph(3´)-I*	-	-
JW691	*aph(3´)-I*	-	-
JW712	*aph(3´)-I*	-	-
JW719	*aph(3´)-IIb*	-	-
JW738	*aph(3´)-I*	-	-
JW751	*aph(2´´)-Ib*	-	-
JW790	*aph(2´´)-Ib*	-	-
JW841	-	bla_{NDM}	-
JW861	*ant(3´´)-I*	-	-
JW874	*ant(3´´)-I*	-	-
JW879	*aph(2´´)-Ib*	-	-
JW910	*aph(4´)-Ia*	-	-

JW921	-	-	*bla*$_{CTX-M}$
JW932	*ant(4´)-Ia*	-	-
JW946	-	*bla*$_{VIM}$	-
JW955	*aph(2´´)-Ib*	-	-
JW961	-	-	*bla*$_{CTX-M}$
JW972	*ant(4´)-Ia*	-	-
JW988	-	*bla*$_{NDM}$	-
JW991	*ant(3´´)-I*	-	-
JW998	*aph(2´´)-Ib*	-	-

Supplementary figure S1: Distribution of AME genes in test isolates

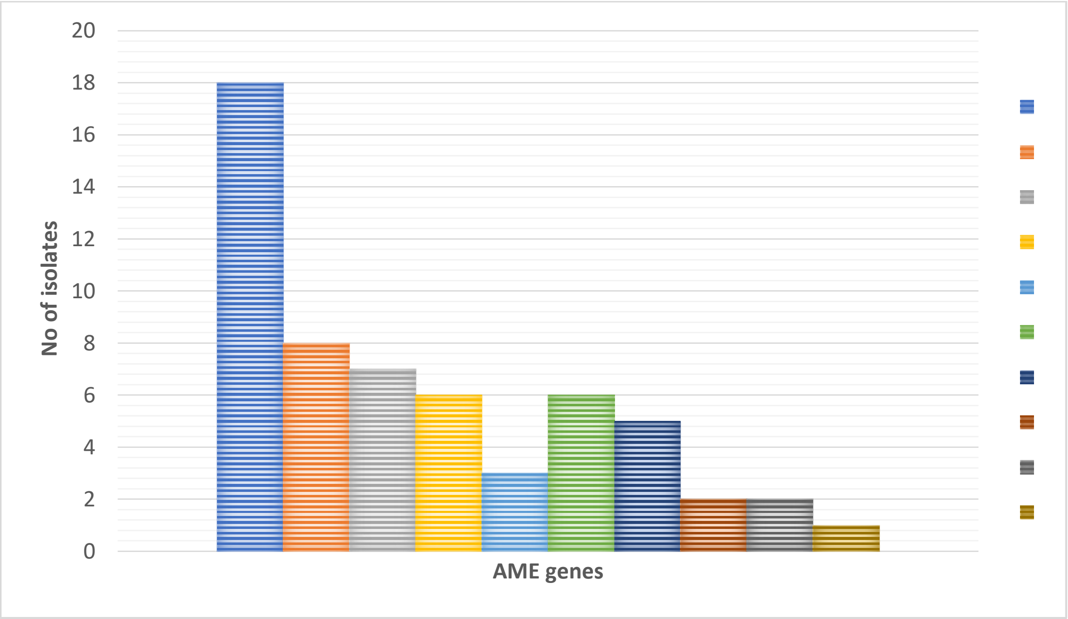

Supplementary Table S5: Zone of inhibition of clones towards aminoglycosides

Sl. No	Clones	Zone of inhibition		
		**Amikacin (Zone size in mm)**	**Gentamicin (Zone size in mm)**	**Tobramycin (Zone size in mm)**
1	Clone 1	16 mm	13 mm	14 mm
2	Clone 2	16 mm	13 mm	14 mm
3	Clone 3	15 mm	14 mm	13 mm
4	Clone 4	16 mm	14 mm	14 mm
5	Clone 5	15 mm	14 mm	14 mm
6	Clone 6	16 mm	13 mm	13 mm
7	Clone 7	16 mm	13 mm	13 mm
8	Clone 8	16 mm	14 mm	14 mm
9	Clone 9	15 mm	14 mm	13 mm
10	Clone 10	15 mm	13 mm	13 mm
